# Total cholesterol to high-density lipoprotein cholesterol ratio is a significant predictor of nonalcoholic fatty liver: Jinchang cohort study

**DOI:** 10.1186/s12944-019-0984-9

**Published:** 2019-02-11

**Authors:** Xiao Yu Ren, Dian Shi, Jiao Ding, Zhi Yuan Cheng, Hai Yan Li, Juan Sheng Li, Hong Quan Pu, Ai Min Yang, Cai Li He, Jian Ping Zhang, Yu Bao Ma, Ya Wei Zhang, Tong Zhang Zheng, Ya Na Bai, Ning Cheng

**Affiliations:** 1 Key Laboratory of Preclinical Study for New Drugs of Gansu Province, Basic Medical College, Lanzhou University, Lanzhou, Gansu 730000 People’s Republic of China; 2 Institute of Epidemiology and Statistics, School of Public Health, Lanzhou University, Lanzhou, 730000 Gansu People’s Republic of China; 3 Workers’ Hospital of Jinchuan Group Co., Ltd., Jinchang, Gansu China; 4 Department of Epidemiology, School of Public Health, Brown University, City of Providence, RI USA; 5 Department of Surgery, School of Medicine, Yale Cancer Center, Yale University, City of New haven, CT USA

**Keywords:** High-density lipoprotein cholesterol, Jinchang cohort, Nonalcoholic fatty liver, Predictive value, Total cholesterol

## Abstract

**Background:**

Some studies found out that TC/HDL-C ratio is a predictor of Cardiovascular disease (CVD) and Nonalcoholic fatty liver (NAFLD) is related to CVD. And some researches have already studied that Apolipoprotein B to Apolipoprotein A1 ratio (ApoB/ApoA1) and Triglyceride to High-density lipoprotein cholesterol ratio (TG/HDL-C) were both related with CVD and NAFLD, but few studied the association between TC/HDL-C ratio and NAFLD. So, we suspected the ratio was also related to NAFLD. The research aims to study the predictive value of TC/HDL-C to NAFLD and to help the early detection of NAFLD.

**Methods:**

Based on the Jinchang Cohort, the study contained 32,121 participants. We assessed the incidence of NAFLD by the quartiles of TC, HDL-C and TC/HDL-C. Then, the does-response relationship between these indicators and the risk of NAFLD was obtained. Finally, the receiver operator characteristic curve (ROC) was applied to decide the predictive value of TC/HDL-C.

**Results:**

Among the study participants, the cumulative incidence of NAFLD was 6.30% and the rate of dyslipidemia was 40.37%. The biochemical indicators of NAFLD had a difference with general population. The incidence of NAFLD raised with the quartiles of TC, TG and LDL-C raising, while decreased with the HDL-C′ quartiles raising. After controlling confounding factors, TC and TC/HD-C had a positive relationship with NAFLD, while HDL-C had the opposite. Finally, the ROC analysis showed the area under the curve (AUC) of TC/HDL-C (0.645) was greater than TC (0.554), HDL-C (0.627) and Apolipoprotein B to Apolipoprotein A1 (ApoB/ApoA1) (0.613).

**Conclusions:**

The TC/HDL-C ratio has significant predictive value to NAFLD.

**Electronic supplementary material:**

The online version of this article (10.1186/s12944-019-0984-9) contains supplementary material, which is available to authorized users.

## Introduction

Recently, NAFLD has been the most common chronic liver disease worldwide [1]. NAFLD is an important problem which can affect the public health all over the world; in both adults and children, it’s accompanied high rates of Cardiovascular disease (CVD). Meanwhile, NAFLD has a relationship with insulin resistance and hyperglycaemia, thus, it’s closely linked to type 2 diabetes [2, 3].

NAFLD is a global issue worldwide. In western countries such as North America, Europe and Australia, NAFLD has been the main chronic liver disease [4, 5]. It has been estimated that the prevalence of NAFLD varies between 20 and 30% among the western countries, Middle East and Japan. India subcontinent is 16–32% while China has a similar prevalence of 15–30% [6, 7]. Thus, it’s an important task for identifying the future development of NAFLD in the population.

A few researches have indicated that the ratio of ApoB/ApoA1 is a predict factor of NAFLD [8], and the ratio is also associated with metabolic syndrome and CVD [9–11]. TC/HDL-C is the most important determining factor of high-sensitivity C-reactive protein (hs-CRP), and hs-CRP is a biomarker of CVD risk, so TC/HDL-C can also predict CVD [12, 13]. Besides, the Framingham Cardiovascular Institute suggested that TC/HDL-C should be <4-to-1 [14]. Several studies also proved the ratio also had relationship with coronary heart disease [15, 16]. Because both of the ratio and NAFLD have relationships with CVD and on the grounds of the biological evidence summarized above, we hypothesized that TC/HDL-C might predict incident NAFLD. The study aims to identify the population which is at risk for future development of NAFLD. So based on the Jinchang Cohort, which has already studied the all causes of death, especially for cancer and cardiovascular diseases [17–20], we investigate the association between TC/HDL-C and NAFLD.

## Methods

### Participants

**Fig. 1:**
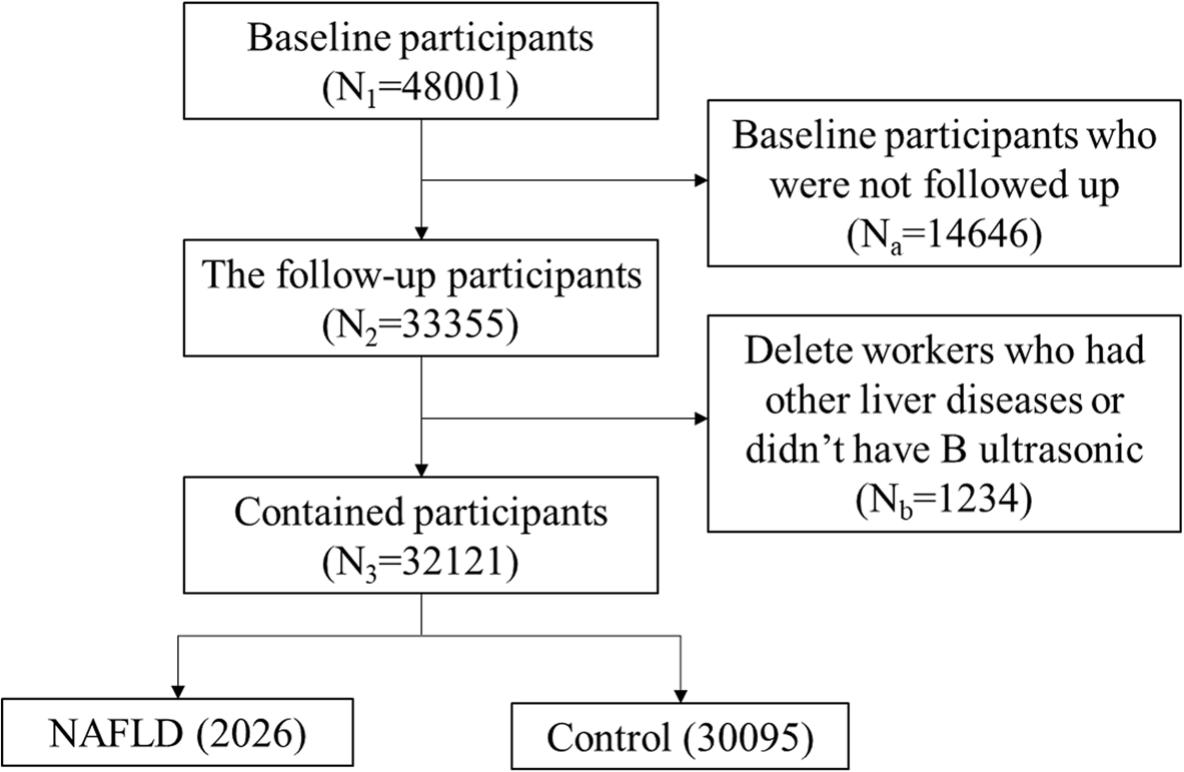
Inclusion and exclusion of the study

The research was based on Jinchang Cohort in Jinchang city, Gansu province, China. The cohort comes from a non-ferrous metals and chemical joint enterprise. The design and methods of the Jichang Cohort Study have previously been described in detail, so we just briefly introduced the cohort [21, 22]. Jichang Cohort was established by a baseline survey from June 2011 to December 2013 which contained in-person interview, physical exams, lab-based tests and biosample collection of all 48,001 participants. From December 2013 to December 2015, the cohort has finished its first follow-up and 33,355 workers were followed up. After deleting 1234 workers who had other liver diseases or didn’t have B ultrasonic results, the study contained 32,121 workers at last. Figure 1 was the structure of the participants in or out. Consent was obtained from each participant included in the study and the study was approved by the Ethics Committee of JNMC.

### Epidemiology survey

Questionnaire data was obtained from personal interviews including the general demographic characteristics, lifestyle, dietary habits, the occupational history, the history of diseases, the family history and the female reproductive history. All the processes of data collection were under strict quality control: Investigators were professional trained, and the auditors checked the questionnaires strictly to find out the mistakes and corrected them. Afterwards, the professional keyboarders used Epi Data3.0 software to record the questionnaires by double blind, and meanwhile, they corrected logical errors to make sure the authenticity of the data.

### Physical examination

The examination included height, weight, blood pressure, B ultrasonic, electrocardiogram and Chest X-ray and this study mainly used B ultrasonic. The diagnosis of NAFLD was in accordance with the NAFLD diagnostic criteria developed by European Association for the Study of the Liver (EASL) [23, 24]. NAFLD diagnosed in this study must has 3 points as follows at the same time:

1. Don’t have the history of drinking or the amount of alcohol< 30 g/d for men and < 20 g/d for women.
2. Despite the population with a history of specific diseases which can result in NAFLD, including virus hepatitis (HBV and HCV), Wilson disease, haemochromatosis and autoimmune hepatitis.
3. The B ultrasonic shows that excessive hepatic fat accumulation and the presence of steatosis in >5% of hepatocytes [23].

### Laboratory examination

All participants provided blood samples, and 3 tubes of fasting venous blood were collected and were put respectively into centrifuge tubes which contained anticoagulant and no anticoagulant. The examined indexes included TG, TC, HDL-C, LDL-C and so on. The 7600–020 automatic biochemical analyzer which was made by Japan Hitachi Company was used for biochemical test of blood. Triglycerides test kits (1555-40-2009), High density lipoprotein cholesterol test kits (0967-40-2009) and Low density lipoprotein cholesterol test kits (0972-40-2009) which were produced by Shanghai Rongsheng biological pharmaceutical Co., LTD were used to test TG, HDL-C and LDL-C. Total cholesterol test kits (1555-40-2009) which were produced by Shanghai Kehua bio-engineering Co., LTD were used to test TC.

### Confirming of the TC/HDL-C ratio

First, the study gave a brief description of physiological and biochemical indicators at baseline in the Jinchang Cohort and compared the indicators between NAFLD and non-NAFLD group. Then, under the different levels of TC, HDL-C and TC/HDL-C, the relationships between these indicators and the incidence of NALFD were analyzed. After that, based on prospective cohort study, we assessed the effect of these three indicators to the risk of NAFLD. Finally, we analyzed TC/HDL-C, TC, HDL-C and ApoB/ApoA1 with ROC, and compared the AUC to estimate the most valuable indicator.

### Statistical analysis

We used SPSS21.0 to conduct the general statistical descriptions, to do Student’s t-test to compare the means of different indicators, and to do Pearson chi-square test to compare the different ratios. Significance was set at α = 0.05. SAS9.3 was used to carry out restricted cubic spline regression (RCS) analysis and the MedCalc 15.8 was used to analyze the data with ROC.

## Results

### The incidence of NAFLD in the Jinchang cohort

Table 1 shows, the new cases of NAFLD were 2026 during the two years of follow up, and the cumulative incidence of NAFLD was 6.30%. The rate of dyslipidemia was 40.37% in the cohort, including TC 6.85%, TG 35.55%, HDL-C 5.56% and LDL-C 6.54%.

**Table 1:** The incidence of NAFLD and the positive rate of dyslipidemia

Metabolic indicators	Reference range	Total	Abnormal	Incidence (%)
NAFLD	–	32,121	2026	6.30
dyslipidemia^†^	–	32,121	12,968	40.37
TC(μmol/L)	<6.10	32,121	2201	6.85
TG(μmol/L)	<1.82	32,121	11,418	35.55
HDL-C(μmol/L)	>0.90	32,121	1785	5.56
LDL-C(μmol/L)	<4.11	32,121	2100	6.54

^†^Dyslipidemia includes high TC, high TG, low HDL-C and elevated LDL-C

*NAFLD* Nonalcoholic fatty liver, *TC* Total cholesterol, *TG* Triglyceride, *HDL-C* High-density lipoprotein cholesterol, *LDL-C* Low-density lipoprotein cholesterol

In Table 2, the physiological and biochemical indicators of NAFLD had a difference with the control group. The average age of NAFLD population was 48.36 years old which was higher than the control group and the same with BMI. TC (4.66 mmol/L), TG (2.58 mmol/L), LDL-C (3.08 mmol/L) of NAFLD population were higher than control group, while HDL-C (1.21 mmol/L) was lower than the control group.

**Table 2:** Comparison of indexes between NAFLD and normal population(X ± S)

Characteristics	Normal(*n* = 30,095)	NAFLD(*n* = 2026)	*T*	*P*
Age(years)	46.58 ± 13.88	48.36 ± 13.53	−10.32	<0.01
BMI(kg/m^2^)	23.07 ± 3.12	26.25 ± 4.04	− 77.32	<0.01
TC(mmol/L)	4.66 ± 0.89	1.92 ± 0.96	−23.29	<0.01
TG(mmol/L)	1.78 ± 1.39	2.58 ± 1.81	−43.87	<0.01
HDL-C(mmol/L)	1.41 ± 0.36	1.21 ± 0.29	44.92	<0.01
LDL-C(mmol/L)	2.97 ± 0.78	3.08 ± 0.86	−10.93	<0.01

*NAFLD* Nonalcoholic fatty liver, *BMI* Body Mass Index, *TC* Total cholesterol, *TG* Triglyceride, *HDL-C* High-density lipoprotein cholesterol, *LDL-C* Low-density lipoprotein cholesterol

### The effect of TC, HDL-C and TC/HDL-C to the incidence of NAFLD

Table 3 shows the relationships between the quartiles of TC, HDL-C and TC/HDL-C these three indexes with the incidence of NAFLD. With the quartiles of TC and TC/HDL-C raising, the incidence of NAFLD raised as well and the trend had statistical significance. However, the incidence of NAFLD decreased because of the HDL-C′ quartiles raising and the trend had statistical significance as well.

**Table 3:** Different metabolic indexes and the ratios with the incidence of NAFLD

Variable	Q1		Q2		Q3		Q4		χ^*2*^	*p*
	N	n(%)	N	n(%)	N	n(%)	N	n(%)		
TC	9130	374 (4.10)	6923	368 (5.32)	7577	559 (7.38)	8491	725 (8.54)	173.25	< 0.01
HDL-C	9214	642 (6.97)	7723	587 (7.60)	7580	471 (6.21)	7604	326 (4.29)	81.28	< 0.01
TC/HDL-C	9526	228 (2.39)	7450	410 (5.50)	7627	651 (8.54)	7518	737 (9.80)	474.62	< 0.01

*TC* Total cholesterol, *HDL-C* High-density lipoprotein cholesterol, *TC/HDL-C* Total cholesterol/ High-density lipoprotein cholesterol

### Dose-response relationship of TC, HDL-C, TC/HDL-C and the risk of NAFLD

**Fig. 2:**
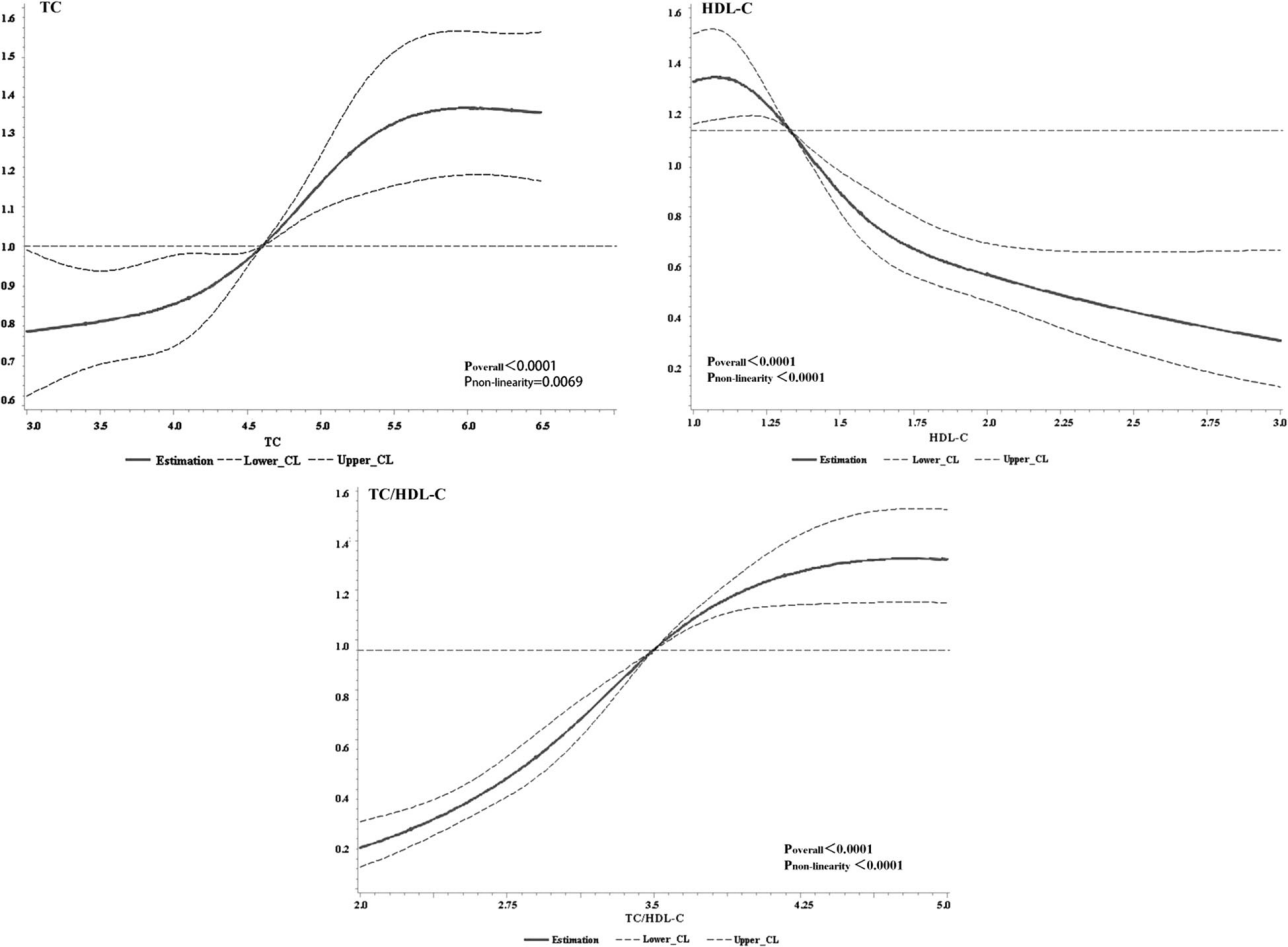
Dose-response relationship of TC, HDL-C, TC/HDL-C and the risk of NAFLD. By using RCS analysis, Cox regression model was applied to evaluate the Hazard Ratio (HR) of NAFLD. After adjusting sex, age, profession and education these possible confounding factors (See details in Additional file 1: Table S1), the HR of NAFLD had non-linearity positive relationship with TC/HDL-C (P_overall_<0.0001, P_non-linearity_<0.0001**)** and TC (P_overall_<0.0001, P_non-linearity_ = 0.0069**)**, non-linearity negative relationship with HDL-C (P_overall_<0.0001, P_non-linearity_<0.0001**)**. HR, Hazards Ratio; TC, Total cholesterol; HDL-C, High-density lipoprotein cholesterol; TC/HDL-C, Total cholesterol/ High-density lipoprotein cholesterol

Figure 2 showed the results of the relationships between lipid metabolisms and the risk of NAFLD by using RCS analysis. After adjusting the confounding factors (see Additional file 1: Tables S1 and S2), we found TC, HDL-C and their ratio had does-response relationship with the risk of NAFLD (Poverall<0.0001). TC had the positive relationship with NAFLD while HDL-C had the opposite. As for the ratio, TC/HDL-C had the positive relationship with NAFLD.

### The ROC analysis of the ratio and the risk of NAFLD

**Fig. 3:**
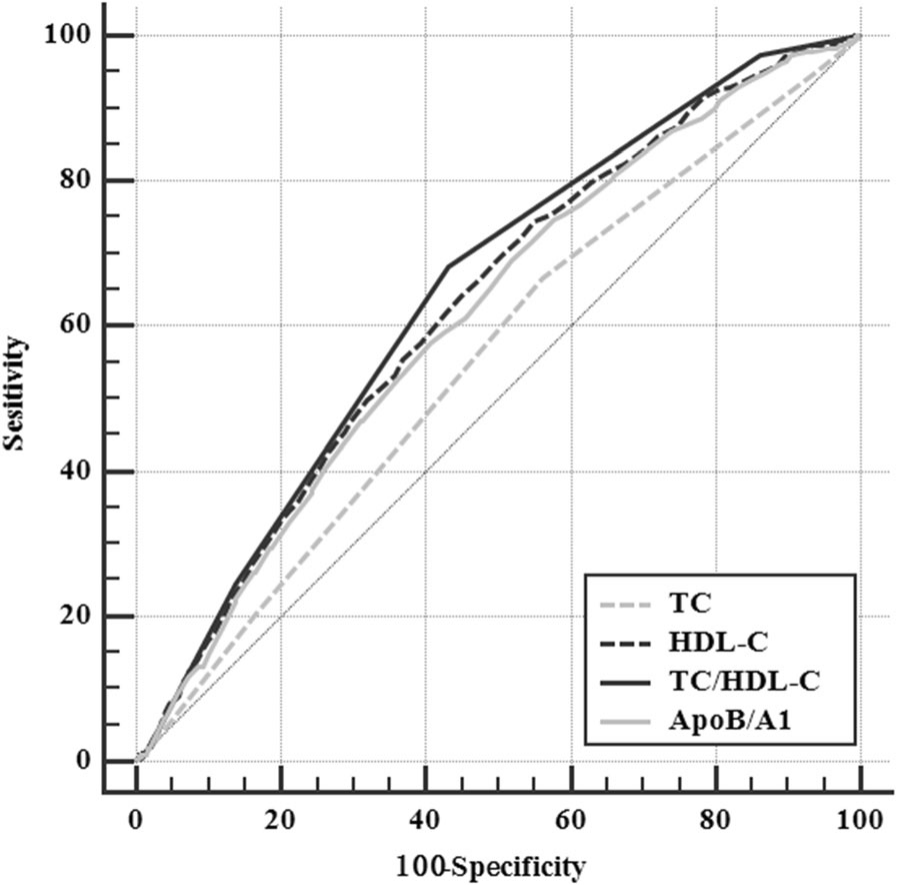
The ROC of TC, HDL-C, TC/HDL-C and ApoB/ApoA1 for NAFLD. The area under the ROC curve (AUC) shows the ability of lipid parameters, including TC, HDL-C (we used the inverse HDL-C instead of HDL-C, because HDL-C had a negative relationship with the risk of NAFLD). TC/HDL-C and ApoB/ApoA1 for the incident of NAFLD. The AUC of TC/HDL-C (0.645 [95%Cl 0.636–0.653]) was greater than that of TC (0.554 [95%Cl 0.545–0.563], *P*<0.0001), that of HDL-C (0.627 [95%Cl 0.618–0.635], *P* = 0.0292) and that of ApoB/ApoA1 (0.613 [95%Cl 0.605–0.622], *P* = 0.0003). TC, Total cholesterol; HDL-C, High-density lipoprotein cholesterol; TC/HDL-C, Total cholesterol/ High-density lipoprotein cholesterol; ApoB/ApoA1, Apolipoprotein B/ Apolipoprotein A1

Figure 3 was the ROC of TC, HDL-C, TC/HDL-C and ApoB/ApoA1. As the figure showed, the AUC of TC/HDL-C was greater than TC, HDL-C and ApoB/ApoA1. This indicated that the predictive value of TC/HDL-C was higher than any of these indexes.

## Discussion

In this cohort study, we found the cumulative incidence of NAFLD in the Jinchang Cohort was 6.30% after two years following up, and the abnormal rate of serum lipid indexes in the cohort was higher than the control group. We also found the lipid indexes were associated with incidence of NAFLD, especially for TC/HDL-C. The AUC of TC/HDL-C for incident NAFLD was greater than those of TC and HDL-C, and also greater than that of ApoB/ApoA1.

There have been researches showed that low levels of HDL-C was caused by lacking of exercises, obesity and diabetes and all of these three factors were risk factors of NAFLD. Souza has pointed out that low levels of HDL-C was the risk factors of NAFLD [25, 26]. Some studies also mentioned TC was a dangerous factor of NAFLD [27]. Besides, NAFLD severity was associated with the risk of CVD and CHD [28–30], and higher TC/HDL-C ratio was a predictive factor of CVD and CHD [12, 15]. So, we hypothesized that in a way, TC/HDL-C might have connections with NAFLD. This article was based on the large sample prospective cohort to study the predictive value of TC/HDL-C to NAFLD.

The study found that under the different levels of TC, HDL-C and TC/HDL-C, the incidence of NAFLD was different as well in the population. With the levels of TC raising, the incidence of NAFLD raised as well, which was similar to the study conducted by Ballestri [27] However, the levels of HDL-C had the opposite effect with the incidence of NAFLD, and the Framingham Cohort as well as Naim Alkhouri [31, 32] had the similar conclusions.

Many studies were the qualitative researches, and few of them did the quantitative researches. So, based on the cohort study, the article conducted a quantitative research and found out that TC, HDL-C and their ratio had dose-response relationship with the risk of NAFLD. TC/HDL-C had the positive does-response relationship with NAFLD which was same as TC while HDL-C had the opposite. However, this study wanted to know whether TC/HDL-C had predictive value to NAFLD, so next, the study conducted ROC analysis.

Through ROC analysis, we found TC/HDL-C had better predictive value than TC and HDL-C. Many studies have already revealed ApoB/ApoA1 was a diagnostic index of NAFLD [33], so we compared the predictive value of TC/HDL-C with it. The study found that both ApoB/ApoA1 and TC/HDL-C had the predictive value of NAFLD, but the results showed that TC/HDL-C was more significant than ApoB/ApoA1. So in Jinchang Cohort, compared with TC, HDL-C and ApoB/ApoA1, TC/HDL-C has the best predictive value to NAFLD, and maybe it will be a new index to predict NAFLD.

There are some limitations in our article. First, the article was based on the population survey, which might result in the problem of the individual data accuracy. Second, Considering the space of the paper, we did not analyze men and women separately. Last, because of various reasons, we could not test what we found in the vitro experiments. So, we need to explore in the future.

This finding suggested that using TC/HDL-C ratio is helpful for identifying NAFLD, and maybe helpful for CVD and other diseases.

## Conclusions

Higher TC/HDL-C ratio is associated with a greater risk for NAFLD. And we find that the ratio has a significant predictive value for NAFLD. However, extensive studies should be carried out for a further instruction.

## Additional file

Additional file 1: **Table S1.** Relative Risk of some factors in normal and NAFLD groups. **Table S2.** Variable scoring list of Logistic(Cox) regression. (DOCX 26 kb)

## Abbreviations

ApoA1: Apolipoprotein A1
ApoB: Apolipoprotein B
AUC: Area under the curve
BMI: Body Mass Index
CVD: Cardiovascular disease
EASL: European Association for the Study of the Liver
HBV: Hepatitis B Virus
HCV: Hepatitis C Virus
HDL-C: High-density lipoprotein cholesterol
hs-CRP: High-sensitivity C-reactive protein
JNMC: Jinchuan Nonferrous Metals Corporation
LDL-C: Low-density lipoprotein cholesterol
NAFLD: Nonalcoholic fatty liver Disease
NHFPC: National Health and Family Planning Committee of China
RCS: Restricted cubic spline regression
ROC: Receiver operator characteristic curve
TC: Total cholesterol
TG: Triglyceride
