# Supplementary material for: Total cholesterol to high-density lipoprotein cholesterol ratio is a significant predictor of nonalcoholic fatty liver: Jinchang cohort study
Source: Lipids Health Dis. 2019 Feb 11;18:47. doi: 10.1186/s12944-019-0984-9 (PMC6371543; doi:10.1186/s12944-019-0984-9)
Supplement: Supplementary file 1 — Table S1. Relative Risk of some factors in normal and NAFLD groups. Table S2. Variable scoring list of Logistic(Cox) regression. (DOCX 26 kb) [file 12944_2019_984_MOESM1_ESM.docx]

**Additional file**

**Table S1 Relative Risk of some factors in normal and NAFLD groups**

| Variable |  | Dividing |  | Normal  (30095) |  | NAFLD  (2026) |  | OR(95%Cl) |
| --- | --- | --- | --- | --- | --- | --- | --- | --- |
|  |  |  |  |  |  |  |  |  |
| Sex |  | Male |  | 18076 |  | 1137 |  | 1 |
|  |  | Female |  | 12019 |  | 889 |  | 0.83(0.75,0.91) |
| Age |  | ＜40 |  | 10042 |  | 412 |  | 1 |
|  |  | ＜50 |  | 11199 |  | 717 |  | 0.56(0.45,0.69) |
|  |  | ＜60 |  | 3677 |  | 361 |  | 0.87(0.71,1.06) |
|  |  | ＜70 |  | 3620 |  | 421 |  | 1.33(1.07,1.65) |
|  |  | ≥70 |  | 1557 |  | 115 |  | 1.58(1.27,1.95) |
| Education |  | Under the Primary |  | 3692 |  | 369 |  | 1 |
|  |  | Junior |  | 7496 |  | 580 |  | 1.90(1.66,2.18) |
|  |  | Senior |  | 8523 |  | 531 |  | 1.47(1.30,1.66) |
|  |  | College or above |  | 10384 |  | 546 |  | 1.19(1.05,1.34) |
| Profession |  | Technicians |  | 1274 |  | 80 |  | 1 |
|  |  | Cadres |  | 4032 |  | 239 |  | 0.46(0.35,0.62) |
|  |  | Workers |  | 23758 |  | 1567 |  | 0.44(0.35,0.54) |
|  |  | Indoor staff |  | 1031 |  | 140 |  | 0.49(0.40,0.58) |
| Income |  | ＜2000 |  | 15169 |  | 1019 |  | 1 |
|  |  | ＜5000 |  | 14456 |  | 973 |  | 0.93(0.65,1.32) |
|  |  | ≥5000 |  | 470 |  | 34 |  | 0.93(0.65,1.33) |
| Smoking |  | Never |  | 17506 |  | 1191 |  | 1 |
|  |  | Ex-smoking |  | 2336 |  | 163 |  | 0.83(0.70,0.98) |
|  |  | Smoking |  | 10253 |  | 672 |  | 1.06(0.90,1.25) |
| Tea |  | Never |  | 15458 |  | 1021 |  | 1 |
|  |  | Ex-drinking |  | 291 |  | 16 |  | 0.94(0.60,1.46) |
|  |  | Drinking |  | 14346 |  | 989 |  | 1.05(0.67,1.64) |
| Exercise |  | Never |  | 4168 |  | 271 |  | 1 |
|  |  | Occasionally |  | 12014 |  | 801 |  | 0.95(0.83,1.09) |
|  |  | Frequently |  | 13913 |  | 954 |  | 0.97(0.88,1.07) |

**Table S2 Variable scoring list of Logistic(Cox) regression**

| Variable | Variable scoring |
| --- | --- |
| Sex | Male=1, Female=2 |
| Age | ＜40=1, 40～49=2, 50～59=3, 60～69=4, ≥70=5 |
| Education | Under the Primary=1, Junior=2, Senior=3, College or above=4 |
| Profession | Technicians=1, Cadres=2, Workers=3, Indoor staff=4 |
| Income | ＜2000=1, 2000～4999=2，≥5000=3 |
| Smoking | Never =1, Ex-smoking =2, Smoking =3 |
| Tea | Never =1, Ex-drinking =2, Drinking =3 |
| Exercise | Never=1, Occasionally =2, Frequently =3 |
